# Extracellular synthesis of silver nanoparticles by the *Bacillus* strain CS 11 isolated from industrialized area

**DOI:** 10.1007/s13205-013-0130-8

**Published:** 2013-04-17

**Authors:** Vidhya Lakshmi Das, Roshmi Thomas, Rintu T. Varghese, E. V. Soniya, Jyothis Mathew, E. K. Radhakrishnan

**Affiliations:** 1 School of Biosciences, Mahatma Gandhi University, PD Hills (PO), Kottayam, 686 560 Kerala India; 2 Plant Molecular Biology, Rajiv Gandhi Centre for Biotechnology, Thycaud (PO), Poojappura, Thiruvananthapuram, 695 014 Kerala India

**Keywords:** Silver nanoparticle, Biosynthesis, Extracellular, *Bacillus* sp.

## Abstract

Biological synthesis of silver nanoparticles using microorganisms has received profound interest because of their potential to synthesize nanoparticles of various size, shape and morphology. In the current study, synthesis of silver nanoparticles by a bacterial strain (CS 11) isolated from heavy metal contaminated soil is reported. Molecular identification of the isolate showed it as a strain of *Bacillus* sp. On treating the bacteria with 1 mM AgNO_3_, it was found to have the ability to form silver nanoparticles extracellularly at room temperature within 24 h. This was confirmed by the visual observation and UV–Vis absorption at 450 nm. Further characterization of nanoparticles by transmission electron microscopy confirmed the size of silver nanoparticles in 42–92 nm range. Therefore, the current study is a demonstration of an efficient synthesis of stable silver nanoparticle by a *Bacillus* strain.

## Introduction

Nanotechnology involving synthesis and applications of nanomaterials is a rapidly growing field with significant applications in various areas (Duran et al. 2005). The attraction of silver nanoparticles (AgNPs) is mainly because of its application in therapeutics, biomolecular detection, catalysis and also as antimicrobial agents (Sadhasivam et al. 2010; Shrivastava et al. 2009; Wei et al. 2008; Christopher et al. 2011). Microbial synthesis of nanoparticles is eco-friendly and has significant advantages over other processes since it takes place at relatively ambient temperature and pressure (Gade et al. 2008; Mukherjee et al. 2008; Wei et al. 2012). As the size and shape of nanoparticles can also be controlled in microbial synthesis (Narayanan and Sakthivel 2010), screening of unexplored microorganisms for AgNPs synthesizing property is very important.

Microbial synthesis of metal nanoparticles can take place either intracellularly or extracellularly (Ahmad et al. 2003, 2007; Jain et al. 2011; Kalishwaralal et al. 2010; Pugazhenthiran et al. 2009; Saifuddin et al. 2009). Intracellular synthesis of nanoparticles requires additional steps such as ultrasound treatment or reactions with suitable detergents to release the synthesized nanoparticles (Babu et al. 2009; Kalimuthu et al. 2008). At the same time extracellular biosynthesis is cheap and it requires simpler downstream processing. This favours large-scale production of silver nanoparticles to explore its potential applications. Because of this, many studies were focussed on extracellular methods for the synthesis of metal nanoparticles (Duran et al. 2005). When the culture supernatant of *Bacillus megaterium* was treated with aqueous solutions of Ag^+^ ions, within few minutes it formed silver nanoparticles (AgNPs) extracellularly (Saravanan et al. 2011). Studies using culture supernatants of bacteria like *Pseudomonas proteolytica, Pseudomonas meridiana, Arthrobacter kerguelensis, Bacillus indicus,* etc., were also proven its property to form extracellular nanoparticles very effectively (Shivaji et al. 2011). Studies on reduction of Ag+ ions to AgNPs by *Staphylococcus aureus* also highlight the potential of extracellular method of nanoparticle formation (Nanda and Saravanan 2009).

Long and continuous microbial interactions with various metals can be greatly expected among bacterial isolates from industrialized area. This provides high possibility of identifying interesting microbial strains with nanoparticle forming properties. In the current study, soil microorganisms from industrialized area were screened for its potential to form silver nanoparticles. The selected strain was subjected to identification by molecular methods which resulted in its identification as a *Bacillus* sp. and very interestingly the isolate was found to have the ability to synthesize silver nanoparticles both intracellularly and extracellularly. The reduction of silver ions was checked by visual inspection as well as by measuring its UV–visible absorption. Further characterization by transmission electron microscopy confirmed the synthesis of stable silver nanoparticles with size range of 42–94 nm.

## Materials and methods

### Isolation of metal-resistant bacteria

Soil samples were collected from heavy metal contaminated areas of Cochin, Kerala, India, and were used as the source material for bacterial isolation. The samples were serially diluted in sterile 0.8 % NaCl and then plated onto nutrient agar (Babu and Gunasekaran 2009). The colonies obtained were further subcultured on nutrient agar supplemented with 1 mM concentration of filter-sterilized AgNO_3_. This was further incubated at room temperature for 48 h and the plates were observed for bacterial growth. The isolated colonies were subcultured and obtained in the form of pure culture and from this one of the strains CS 11 was selected randomly to explore its potential to form silver nanoparticles.

### Molecular identification

Molecular identification of the isolated strain was carried out by 16S rDNA sequence-based method. Total genomic DNA was isolated from a selected strain for PCR. Quality of the isolated DNA was checked by agarose gel electrophoresis and was further quantified using UV–Vis spectrophotometer (Hitachi U5100). The genomic DNA was then used as template for PCR using the primers 16SF (5′-AgA gTTTgA TCM Tgg CTC-3′) and 16SR (5′-AAg gAggTg WTC CAR CC-3′). The PCR was carried out in a total volume of 50 μL containing 50 ng of genomic DNA, 20 pmol of each primer, 1.25 Units of *Taq* DNA polymerase, 200 μM of each dNTPs and 1× PCR buffer as components. The PCR was performed for 35 cycles in a Mycycler™ (Bio-Rad, USA) with the initial denaturation for 3 min at 94 °C, cyclic denaturation for 30 s at 94 °C, annealing for 30 s at 58 °C and extension for 2 min at 72 °C with a final extension of 7 min at 72 °C. After the PCR, the reaction products were analysed by agarose gel electrophoresis. The product was then gel purified and was further subjected to sequencing PCR using the Big Dye Terminator Sequence Reaction Ready Mix (Applied Biosystem). After the reaction, product was purified, precipitated and was used for sequence run in the DNA sequencer ABI 310 Genetic Analyser. The sequence data of 16S rDNA thus obtained was further aligned using BioEdit program. This sequence was then used for BLAST analysis. The 16S rDNA sequence of CS 11 was also used for phylogenetic analysis using neighbor-joining method in MEGA5 (Tamura et al. 2011).

### Synthesis of silver nanoparticles

For silver nanoparticle biosynthesis studies, the selected bacterial isolate was inoculated in to 250-ml Erlenmeyer flask containing 100 ml sterile nutrient broth. The cultured flasks were incubated in a rotating shaker set at 200 rpm for 48 h at room temperature. After this the culture was centrifuged at 12,000 rpm for 10 min. The biomass and supernatant were separated and used separately for the synthesis of silver nanoparticles. The supernatant was used for studying extracellular production of silver nanoparticles by mixing it with filter-sterilized AgNO_3_ solution at 1 mM final concentration. At the same time, bacterial biomass was taken for intracellular synthesis. For this approximately 2 g of wet biomass was resuspended in 100 ml of 1 mM aqueous solution of AgNO_3_ in a 250-ml Erlenmeyer flask. All the reaction mixtures were incubated on rotating shaker (200 rpm) at room temperature for a period of 72 h in light. Heat-killed samples with AgNO_3_ were also incubated along with experimental samples as control. Visual observation was conducted periodically to check for the nanoparticle formation. Further characterization was conducted for nanoparticle generated through extracellular methods.

### Characterization of silver nanoparticle

The optical characteristics of the synthesized silver nanoparticles were analysed using UV–Vis spectrophotometer. For this, nanoparticle containing samples were subjected to absorption analysis at 200–700 nm range using UV–Vis spectrophotometer (Hitachi U5100). The silver nanoparticles synthesized by the supernatant were further subjected to TEM analysis. This was used to get an insight into size and morphology of the formed silver nanoparticles. Samples for TEM analysis were prepared on carbon-coated copper TEM grids. The films on the TEM grids were allowed to stand for 2 min, then extra solution was removed and the grid was allowed to dry prior to measurement. TEM measurements were recorded using a JEOL-JEM-1011 instrument at 80 kV.

## Results

**Fig. 1:**
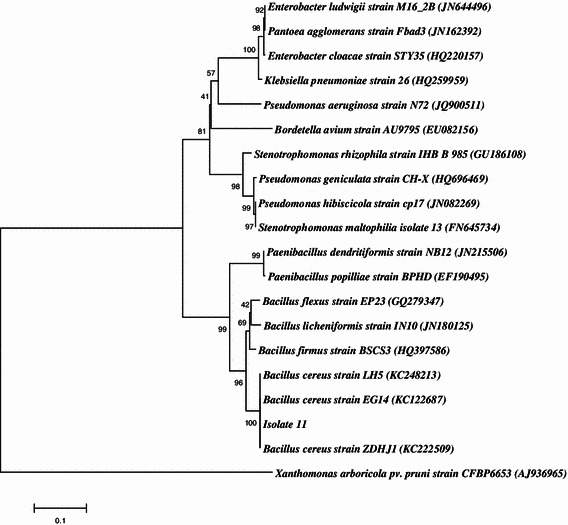
The phylogenetic analysis of the 16S rDNA sequence of the soil bacterial isolate obtained in the study along with other selected sequences from database. The analysis was conducted using neighbor-joining method in MEGA5. Among the various species of *Bacillus*, the isolate formed cluster with *Bacillus cereus*

Among the 11 bacterial isolates (CS 1–CS 11) purified from soil samples, CS 11 was randomly selected for the study and was found to have the ability to form silver nanoparticles as observed by change in colour of the reaction. The selected isolate was further subjected to molecular identification by 16S rDNA sequencing-based method. The sequence data were subjected to BLAST analysis and the result showed its maximum identity of 99 % to various *Bacillus* sp. mainly *Bacillus cereus* (Fig. 1). The 16S rDNA sequence of the isolate was submitted to NCBI under the accession number JN835219.

**Fig. 2:**
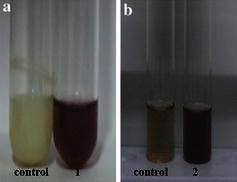
Visual observation of the biosynthesis of silver nanoparticles by biomass and supernatant of *Bacillus* sp. CS 11 selected for the study. **a1** bacterial biomass with AgNO_3_ solution (colour change from *paleyellow* to *brown*) and control heat-killed biomass with AgNO_3_ solution (no colour change). **b2** culture supernatant with AgNO_3_ solution (colour change from *paleyellow* to *brown*) and control heat-killed supernatant with AgNO_3_ solution (no colour change)

The biosynthesis of silver nanoparticles using both biomass and supernatant were separately investigated primarily through the observation of colour change of the experimental samples in the presence of 1 mM AgNO_3_. A colour change from pale yellow to brown occurred for both bacterial biomass and supernatant within 24 h of incubation in the presence of light and is shown in Fig. 2. The positive result as observed by the formation of brown colour was maintained throughout the 72-h period of observation. At the same time, experimental control containing heat-killed biomass or supernatant with silver nitrate showed no colour change. This suggests the colour change observed in the bacterial biomass and the supernatant samples was due to the formation of silver nanoparticles. Even though the colour change was observed for samples containing both biomass and supernatant, further experiments were continued only for the extracellular samples. This is because of the comparative advantages of extracellular synthesis over intracellular method.

**Fig. 3:**
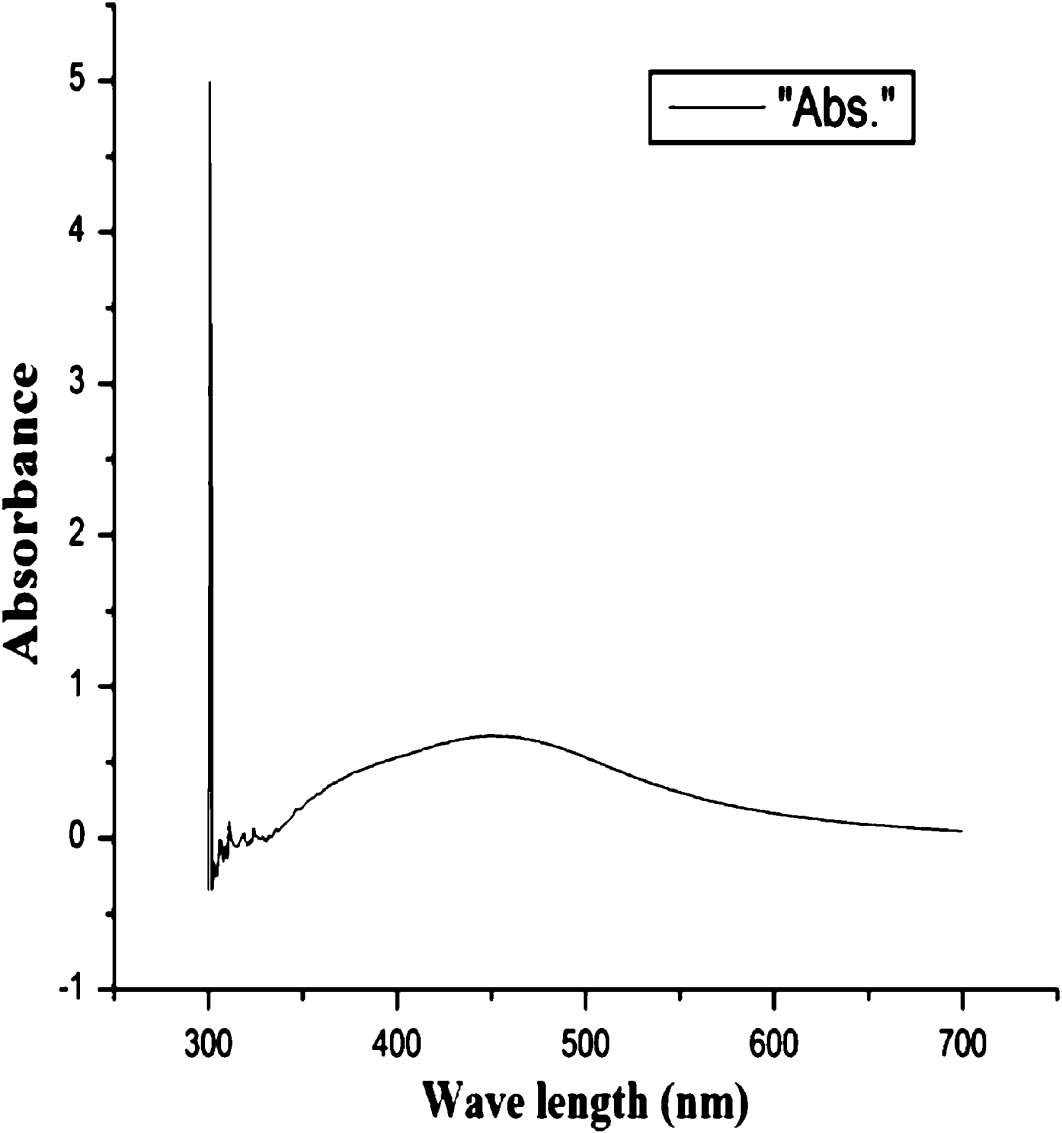
The UV–Vis absorption spectrum of silver nanoparticles synthesized by supernatant of *Bacillus* sp CS 11. The absorption spectrum of silver nanoparticles exhibited a strong broad peak at 450 nm and observation of such a band is assigned to surface plasmon resonance of the particles

The colour change observed for the extracellular samples were further confirmed by UV–Vis spectral analysis as part of primary confirmation. Silver nanoparticles are known to have an intense absorption peak in UV absorption spectra due to its surface plasmon excitation. For the culture supernatant of CS 11, an absorption peak was observed at 450 nm and is an indication of formation of silver nanoparticles (Fig. 3). In addition, these absorption spectra for the silver nanoparticles were obtained within 24 h. The colour change and UV absorption data analysis thus confirms the reduction of AgNO_3_ to silver nanoparticles by the culture supernatant of CS 11.

**Fig. 4:**
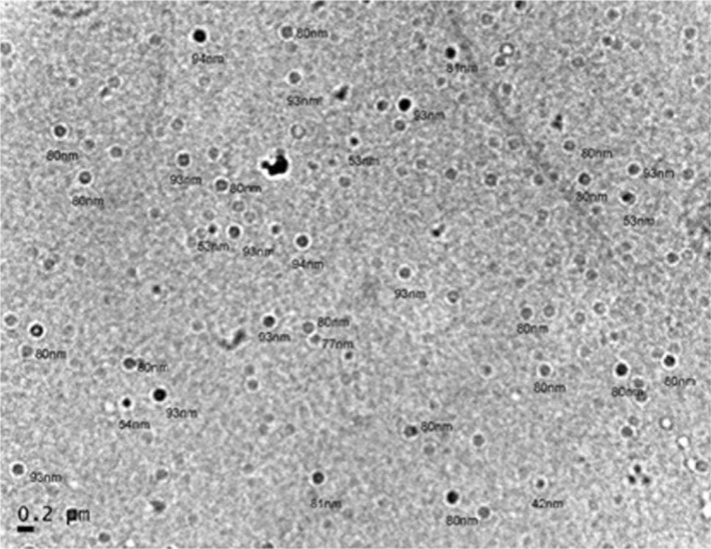
TEM analysis of the silver nanoparticles synthesized by *Bacillus* sp. CS 11 used in the study. TEM image indicates the size controlled synthesis of silver nanoparticles with the particle size ranging from 42 to 94 nm

For further confirmation of nanoparticles formed by bacterial supernatant, the samples were subjected to TEM analysis. The result revealed that the silver nanoparticles were generally spherical in shape and in the size range of 42–94 nm (Fig. 4). The size of the nanoparticles can have direct effect on its physico-chemical properties and this can vary among the particles formed by different groups of microorganisms.

## Discussion

The biological agents in the form of microbes are efficient candidates for the synthesis of nanoparticles. These biogenic nanoparticles are cost-effective, simpler to synthesize and the method is greener in approach. Silver nanoparticle had drawn much attention because of their extensive application to new technologies in chemistry, electronics, medicine and biotechnology. In the current study, a bacterial strain isolated from heavy metal contaminated soil samples was found to have resistance to silver nitrate. Very interestingly, the selected *Bacillus* strain showed the ability to synthesize silver nanoparticles by both extracellular and intracellular synthesis mechanisms. This was observed by the change in colour from pale yellow to brown. Observation on colour change is a method generally used for screening microbial isolates for silver nanoparticle synthesis (Kalimuthu et al. 2008). The excitation of surface plasmon vibration in the silver nanoparticles was considered as the basis for formation of brown colour. Similar observation was previously reported for the supernatant of *Bacillus megaterium*, where a pale yellow to brown colour was formed due to the reduction of aqueous silver ions to silver nanoparticles (Saravanan et al. 2011). This supports the fact that change in colour as observed in the experiment can be considered as an indication of silver nanoparticles formation. This was further confirmed by UV–Vis spectroscopy which measures the absorption spectra of silver nanoparticles formed due to the collective excitation of conduction electrons in the metal. Thus, methods based on UV–Vis spectroscopy have been shown to be an effective technique for the analysis of nanoparticles (Sastry et al. 1998). UV–Vis spectra of silver nanoparticles synthesized by the selected isolate showed an absorption band at 450 nm by treating the supernatant with AgNO_3_. Presence of such peak, assigned to a surface plasmon, was also well documented for silver nanoparticles as reported in the case of *Neurospora crassa* (Longoria et al. 2011).

The mechanism behind the extracellular synthesis of nanoparticles using microbes is not fully known. But it is considered that the enzymes like nitrate reductase secreted by microbes help in the bioreduction of metal ions to metal nanoparticles (Duran et al. 2005). This was reported in *Bacillus licheniformis* where nitrate reductase secreted by the bacteria was found to be responsible for the reduction of Ag^+^ to nanoparticles (Kalimuthu et al. 2008). Detailed analysis of TEM data proved that the silver nanoparticles formed were not in direct contact with each other. This can be taken as a proof of good stabilization of the nanoparticles which can be due to the proteins secreted by microorganisms. The size ranges of silver nanoparticles produced by the CS 11 (42–94 nm) fall closer to the size of silver nanoparticles produced by other bacteria (Gurunathan et al. 2009).

In conclusion, this work demonstrates the silver nanoparticles synthesizing property of bacteria isolated from industrialized area. The isolate CS 11 selected in the study was found to have the potential to form silver nanoparticles extracellularly at room temperature within 24 h. The synthesized silver nanoparticles were characterized by UV–Vis spectroscopy and confirmed by TEM. The nanoparticles formed by the isolate were found to be stable with size range of 42–92 nm which indicate its potential applications.
